# Arthroscopically assisted evaluation of frequency and patterns of meniscal tears in operative tibial plateau fractures: a retrospective study

**DOI:** 10.1186/s13018-021-02265-0

**Published:** 2021-02-06

**Authors:** Xiangtian Deng, Hongzhi Hu, Yuchuan Wang, Decheng Shao, Yingze Zhang

**Affiliations:** 1 School of Medicine, Nankai University, Tianjin, 300071 People’s Republic of China; 2 Department of Orthopedics, The Union Hospital of Tongji Medical College of Huazhong University of Science and Technology, Wuhan, 430022 China; 3 Department of Orthopedics, The Third Hospital of Hebei Medical University, Shijiazhuang, 430022 China

**Keywords:** Frequency of meniscal tears, Tibial plateau fracture, Meniscus tear morphology, Schatzker classification, Arthroscopy

## Abstract

**Background:**

Despite tibial plateau fractures are often associated with meniscal tears, the association between meniscal tears and Schatzker classification remains unclear. The purpose of this study was to assess the frequency and patterns of meniscal tears in operatively treated tibial plateau fractures following immediate arthroscopic evaluation after internal fixation of tibial plateau fractures and to reveal the association between these concomitant meniscal tears and Schatzker classification.

**Methods:**

A total of 252 consecutive patients (166 males and 86 females, mean age 46.7 (19–80) years) with operatively treated tibial plateau fractures admitted to our hospital from January 2016 to May 2019 were performed. Arthroscopic examination for frequency and patterns of meniscal tears was evaluated and documented at the time of surgery, and the association between the frequency and patterns of meniscal tears with Schatzker classification was then analyzed.

**Results:**

The overall frequency of meniscal tears in TPFs was 67% (168 of 252) with 33% (84 of 252) of these being lateral meniscal tears, and 10% (26 of 252) medial meniscal tears, while 23% (58 of 252) had bilateral meniscal tears. Schatzker II was most commonly associated with meniscal tears, occurring in 72% (71 of 99) of our series. There is no significant difference between the frequency of meniscal tears and Schatzker classification (*p* > 0.05). The most common patterns of meniscal tears were longitudinal tears in 23% of tibial plateau fractures (59 of 252), and it occurred at a significantly higher frequency in Schatzker II with 43% (43 of 99). Schatzker IV had significantly higher prevalence of bucket-handle tears than other fracture patterns (*p* < 0.05), and Schatzker VI fractures had significantly higher prevalence of complex tears than other fracture patterns (*p* < 0.05). For other Schatzker classification, the patterns of meniscal tears demonstrated no statistical difference (*p* > 0.05).

**Conclusion:**

The results identified that meniscal tears are commonly seen in each Schatzker classification. Although various patterns of meniscal tears occurred in tibial plateau fractures, the most common patterns were longitudinal tears. Importantly, we suggest that the status of meniscal tears associated with TPFs should be considered at the time of surgery in addition to fracture fixation.

## Background

Meniscal tears are common intra-articular soft tissue injuries associated with tibial plateau fractures (TPFs), resulting in mechanical symptoms such as clicking, locking of the knee joint even earlier onset of post-traumatic knee osteoarthritis when meniscal tears are overlooked [1], which can decrease patients’ satisfactory outcome. Although optimal treatment of these fractures continues to evolve in recent years, the emphasis on the prompt intervention and treatment of the meniscal tears has remained a topic in the management of TPFs.

Tibial plateau fractures, usually attributed to bi-modal injuries caused by high-energy trauma in young patients and low-energy injuries in elderly patients, which consisted of approximately 1.6% of adult fractures in China [2]. In addition to conventional open reduction and internal fixation (ORIF), multiple surgical procedures for the management of TPFs have been proposed in the literature [3–5]. With the use of our published minimally invasive procedure for TPFs, following “bidirectional rapid reductor” assisted closed reduction and internal fixation in TPFs, which have reported that low complication rates and excellent functional recovery [6].

In clinical practice, it is generally accepted that meniscal tears need to be repaired in the context of TPFs to enhance mechanical stability of the knee joint, and to minimize tibiofemoral joint contact pressure [7, 8]. Previous studies with respect to meniscal tears utilizing magnetic resonance imaging (MRI) examinations have demonstrated that the occurrence frequently ranging from 47-99% in conjunction with TPFs [7, 9–11]. Nevertheless, MRI evaluation pre-operatively may not provide exact frequency and patterns of meniscal tears due to the interference of fracture hematoma and the loss of normal anatomy structure in severe comminuted fractures. As is well known, arthroscopic examination is the “gold standard” to evaluate meniscal tears in cases of TPFs, but it has not yet become commonplace in the evaluation of meniscus integrity associated with TPFs due to a massive amount of irrigation’s fluid which may increase the risk of acute compartment syndrome occurrence [12–14].

This retrospective study aimed to analyze the frequency and patterns of meniscal tears with the use of immediate arthroscopic examination following the internal fixation of TPFs. The secondary purpose was to reveal the association between these concomitant meniscal tears and Schatzker classification. Our hypothesis was that arthroscopically assisted evaluation for meniscal tears concomitant with TPFs would reveal a high frequency of meniscal tears in each Schatzker classification. We also hypothesized that the morphology of meniscal tears would have a significant difference of frequency in each Schatzker classification.

## Methods

### Patients

Between January 2016 and May 2019, a total of consecutive 571 patients with TPFs were admitted to our trauma level-1 center of the Third Affiliated Hospital of Hebei Medical University. For this study, the patient inclusion criteria were as follows: (a) tibial plateau fractures confirmed by computer tomography (CT) scans; (b) patients aged older than 18 years; (c) closed fractures; (d) patients who underwent closed reduction and internal fixation combined with arthroscopic examination. The exclusion criteria were as follows: (a) skeletally immature patients at the time of surgery; (b) open or pathological fractures; (c) patient underwent ORIF alone without arthroscopic examination; (d) ipsilateral peri-articular fractures; (e) severe pre-existing knee osteoarthritis; (f) non-operative treatment. The operative indications for TPFs included articular step-off exceeding 3 mm, condylar widening greater than 5 mm, or malalignment greater than 5° [15]. Importantly, arthroscopic examination was performed on all included TPFs patients after fracture fixation.

### Radiological parameters and meniscal tears morphology

**Fig. 1:**
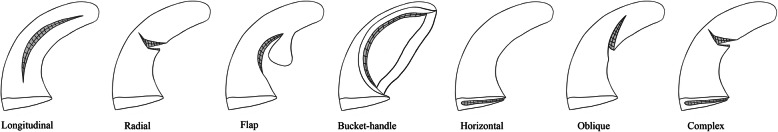
Schematic illustration of classification of meniscal tears

**Fig. 2:**
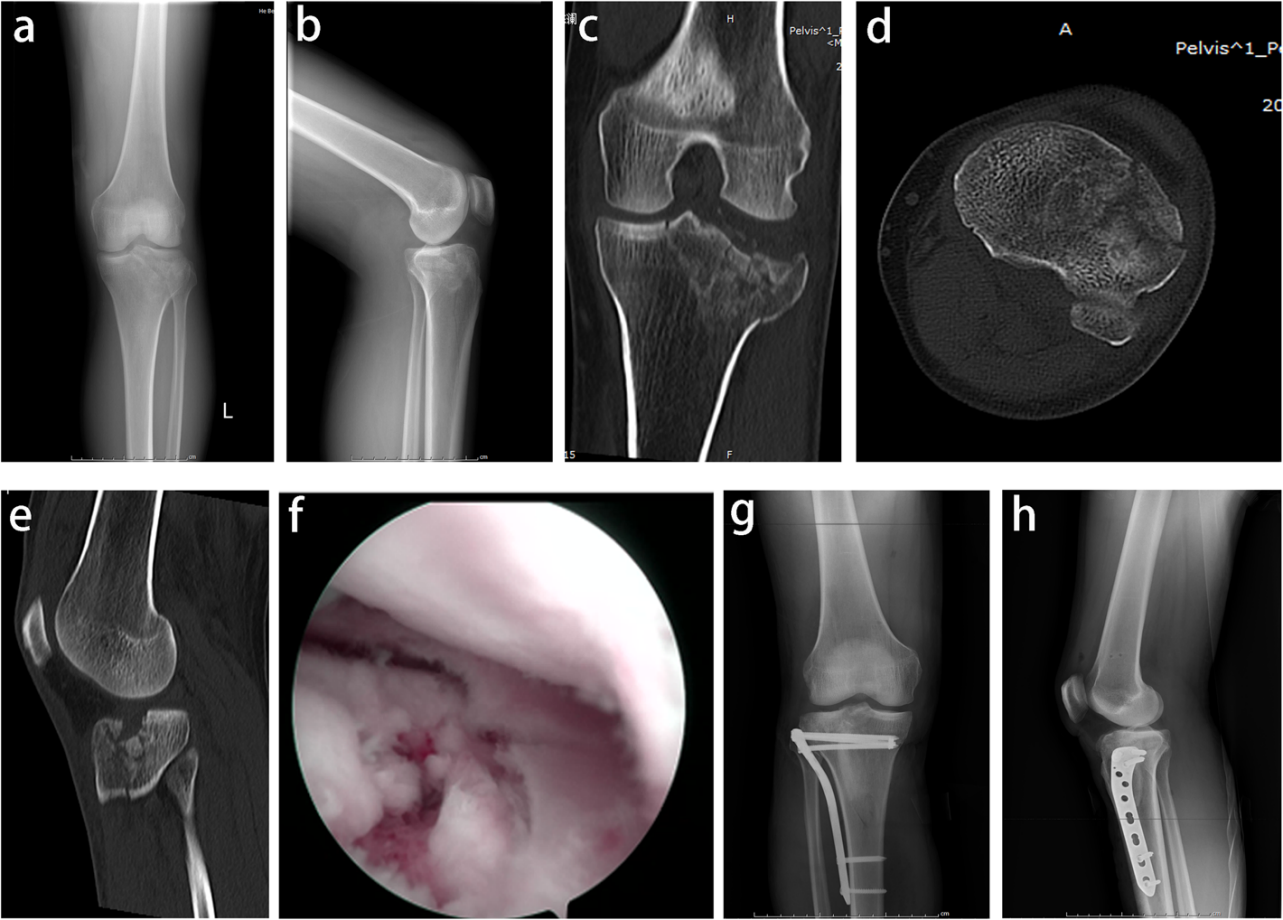
A case of Schatzker type II tibial plateau fractures, male, 47 years old. **a**, **b** Preoperative radiographs of the injured knee. **c** Preoperative coronal CT scan shows the lateral tibial plateau fracture, increased width of the plateau, and articular depression. **d** Axial CT scan shows tibial plateau fracture involved the anterolateral column. **e** Sagittal CT scan shows articular depression concentrated in the anterolateral plateau**. f** Arthroscopic findings intra-operatively shows the radial meniscal tears associated with tibial plateau fracture. **g-h** Post-operative radiographs

All of the patients who underwent both knee radiographs (anteroposterior and lateral views) and CT scans of their injured knees pre-operatively to evaluate fracture patterns. Despite TPFs were classified in accordance with the Schatzker classification [16] according to conventional radiographs, we applied CT scanning to classify these fractures types. Patients’ demographics were based upon Schatzker classification are shown in Table 1. The operative planning and surgical technique have been described in our previously published literature [17]. In addition, all patients had an immediate arthroscopic evaluation at the time of surgery to evaluate the integrity of the meniscus. Arthroscopic examination for frequency and patterns of meniscal tears were documented [18] and its association with each Schatzker classification was then analyzed. Classification of meniscal tears morphology are described as longitudinal tears, radial tears, flap tears, bucket-handle tears, horizontal tears, oblique tears, and complex tears in terms of the geometry configurations of meniscus under arthroscopy examination (Fig. 1). Complex tears were defined as involving multiple planes and are a combination of other patterns including two or more tear patterns. Figure 2 illustrates a patient with meniscal radial tears concomitant with split and articular depression of tibial plateau fracture (Schatzker type II).

**Table 1:** Patient demographics associated with Schatzker classification

Parameters	Schatzker I	Schatzker II	Schatzker III	Schatzker IV	Schatzker V	Schatzker VI	Statistics analysis
Number of fractures (%)	7 (2.7%)	99 (39.3%)^a^	17 (6.7%)	31 (12.3%)	66 (26.2%)	32 (12.7%)	*p* < 0.05
Average age, years (range)	42.0 (29-68)	44.4 (19-75)	38.7 (21-62)	47.7 (25-71)	47.2 (19-80)	51.2 (24-73)	*p* > 0.05
Gender, male/female	5/2	63/36	11/6	21/10	43/23	23/9	*p* > 0.05
Side, right/left	4/3	56/43	13/4	22/9	42/24	18/14	*p* > 0.05
Time from injury to surgery, days (range)	9.9 (3-16)	10.4 (2-30)	10.0 (4-19)	10.7 (3-22)	11.2 (4-25)	11.7 (2-39)	*p* > 0.05

aThere was a statistically significant difference (*p* < 0.05) among Schatzker classification

### Statistical analysis

SPSS software (version 24.0, IBM Corp., USA) was performed for statistical analysis. Analysis of variance, chi-square test, and Fisher’s exact test were used for statistical analysis. For all tests, *P* values < 0.05 were considered statistically significant.

## Results

### Patient demographics

**Fig. 3:**
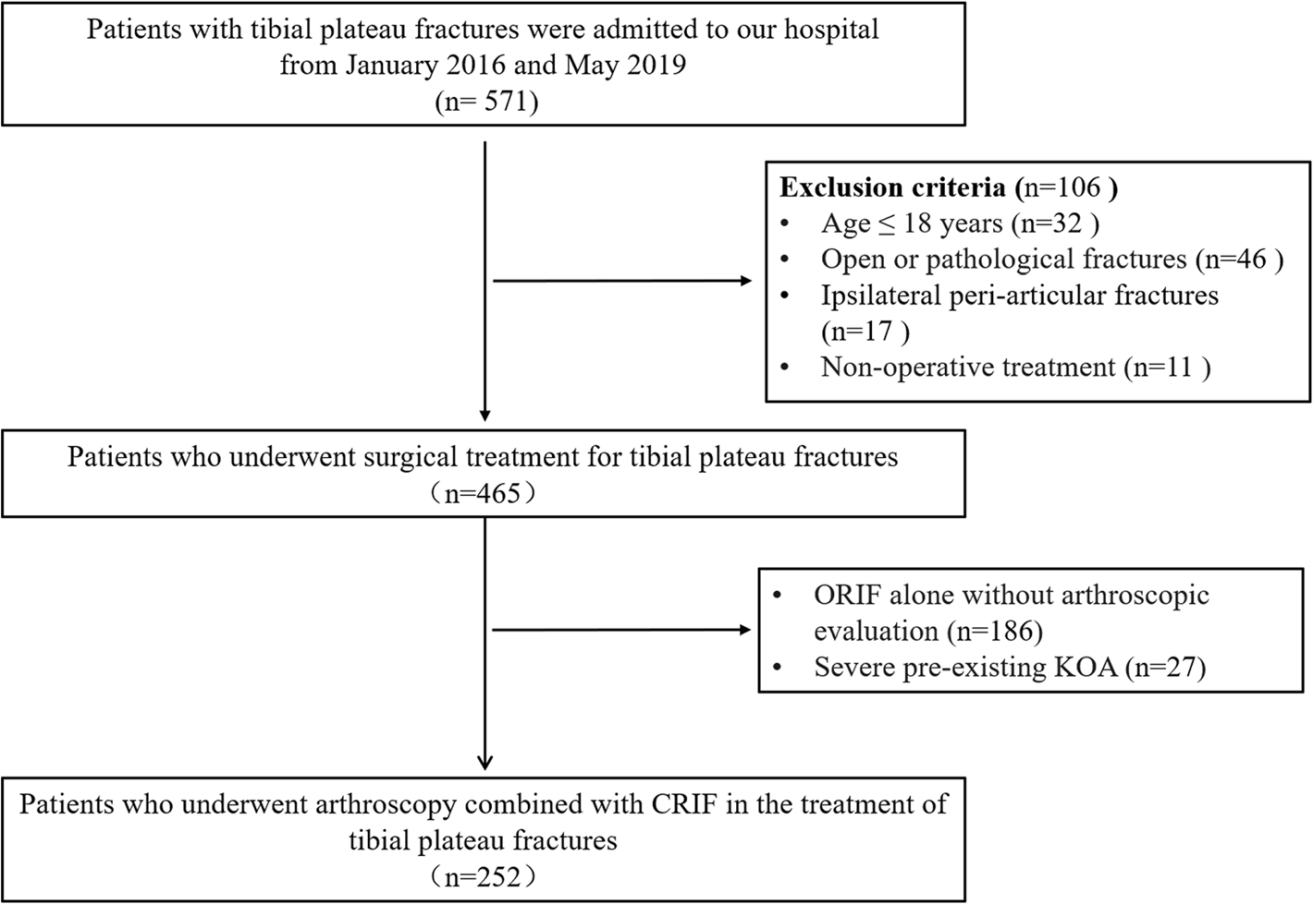
Flow chart showing the steps in patient enrollment. ORIF, open reduction and internal fixation; CRIF, closed reduction and internal fixation

A total of 252 patients (including 166 males and 86 females, with 97 left knee injuries and 155 right knee injuries) of the 571 patients fulfilled these abovementioned criteria and were enrolled in our protocol. Figure 3 showed the flowchart of patient enrollment in this study. The average time from injury to surgery was 10.1 days (range, 2-39 days). The mean age of enrolled patients at the time of operation was 46.7 years (range, 19 to 80 years). The types of injury were as follows: In 108 cases (42.9%) fractures were associated with motor vehicle accident, in 71 cases (28.2%) to falls from heights, in 51 cases (20.2%) to slips-related injury, in 13 cases (5.2%) to twisting-related injury, and in 9 cases (3.6%) of sports injury.

The data of the surgical patients are summarized in Table 1. Our results showed that there was no statistical difference in terms of patients’ demographic based upon Schatzker classification (*p* > 0.05). The distribution of the 252 TPFs according to Schatzker classification was as follows: Schatzker I (split fracture of the lateral plateau without depression) were 7 patients (7/252, 2.7%). Schatzker II (split plus depression of the lateral plateau) were 99 patients (99/252, 39.3%). Schatzker III (pure depression fracture of the lateral plateau) were 17 patients (17/252, 6.7%). Schatzker IV (split and/or depression fracture of the medial plateau) were 31 patients (31/252, 12.3%). Schatzker V (bicondylar plateau fracture) were 66 patients (66/252, 26.2%). Schatzker VI (bilateral plateau fractures associated with metaphysis fracture) were 32 patients (32/252, 12.7%). Furthermore, it needs to be noted that the Schatzker II was the most common TPFs (99/252, 39.3%) which have a significant difference than other Schatzker classification (*p* < 0.05).

### Association between frequency of meniscal tears and Schatzker classification

Table 2 demonstrated the information regarding frequency of meniscal tears based on each Schatzker classification. Of the 252 fractures in our study, 168 (66.7%) patients with TPFs had accompanying meniscal tears through arthroscopic evaluation. Each Schatzker classification had a high frequency of meniscal tears, ranging from 42.8 to 72.7%. According to the meniscal tears in the side of the knee joint, meniscal tears could be described as lateral meniscal tears (LMT), medial meniscal tears (MMT), and bilateral meniscal tears (BMT). The frequency of overall meniscal tears in TPFs was 66.7% (168 of 252) with 33.3% (84 of 252) of these being LMT, and 10.3% (26 of 252) MMT, while 23.0% (58 of 252) had BMT.

**Table 2:** Frequency of affected side of meniscal tears based on Schatzker classification

Parameters	Schatzker I (*n* = 7)	Schatzker II (*n* = 99)	Schatzker III (*n* = 17)	Schatzker IV (*n* = 31)	Schatzker V (*n* = 66)	Schatzker VI (*n* = 32)	Total (*n*, %) (*n* = 252)	Statistics analysis
Meniscal tears (%)	3 (42.8%)	71 (71.7%)	8 (47.1%)	17 (54.8%)	48 (72.7%)	21 (65.6%)	168 (66.7%)	*p* > 0.05
LMT (%)	2 (28.5%)	58 (58.6%)^a^	7 (41.2%)	4 (12.9%)	9 (13.6%)	4 (12.5%)	84 (33.3%)	*p* < 0.05
MMT (%)	0(0%)	5 (5.1%)	0 (0%)	11 (35.5%)^b^	7 (10.6%)	3 (9.4%)	26 (10.3%)	*p* < 0.05
BMT (%)	1 (14.3%)	8 (8.1%)	1 (5.9%)	2 (6.5%)	32 (48.5%)^c^	14 (43.8%)	58 (23.0%)	*p* < 0.05

^a^*p* < 0.05, compared with Schatzker IV, V, VI

^b^*p* < 0.05, compared with Schatzker I, II, III, V, VI

^c^*p* < 0.05, compared with Schatzker II, III, IV

*LMT* lateral meniscal tears, *MMT* medial meniscal tears, *BMT* bilateral meniscal tears

Schatzker II was most commonly associated with overall meniscal tears, occurring in 7.7% (71 of 99) of our series. Moreover, LMT was the highest frequency of meniscal tears which was observed in 84 TPFs and was most frequent in Schatzker II. MMT occurred in 26 (10.3%) of fractures, and in 11 of the 31 (35.5%) of the Schatzker IV. Meanwhile, statistical analysis revealed that Schatzker IV had a significantly higher frequency of MMT as compared to all other fracture patterns (*p* < 0.05). Injury to the bilateral meniscus occurred most frequently in Schatzker V (32 of 66; 48.5%), which was statistically significant (*p* < 0.05). However, there was no significant difference between frequency of overall meniscal tears and Schatzker classification (*p* > 0.05). That is, meniscal tears associated with TPFs are commonly seen in each Schatzker classification.

### Association between meniscal tears morphology and Schatzker classification

The patterns of meniscal tears varied significantly with respect to each Schatzker classification (*p* < 0.05). For TPFs based on Schatzker classification, our study demonstrated that longitudinal tears were the most common patterns of meniscal tears which was observed in 59 (23.4%) of 252 TPFs and was most frequent in Schatzker II. Furthermore, Schatzker IV had significantly higher rate of bucket-handle tears than other fracture patterns (*p* < 0.05). Schatzker VI had significantly higher rate of complex tears than other fracture patterns (*p* < 0.05). For other Schatzker classification, the patterns of meniscal tears demonstrated no statistical difference (*p* > 0.05). Table 3 lists the association between patterns of meniscal tears and each Schatzker classification.

**Table 3:** Relationship between patterns of meniscal tears with Schatzker classification

Patterns of meniscal tears	Schatzker I (*n* = 7)	Schatzker II (*n* = 99)	Schatzker III (*n* = 17)	Schatzker IV (*n* = 31)	Schatzker V (*n* = 66)	Schatzker VI (*n* = 32)	Frequency of meniscal tears	Statistics analysis
Longitudinal (%)	2 (28.6%)	43 (43.4%)^a^	3 (17.6%)	3 (9.7%)	5 (7.6%)	3 (9.4%)	59 (23.4%)^d^	*P* < 0.05
Radial (%)	0 (0%)	5 (5.1%)	2 (11.8%)	3 (9.7%)	6 (9.1%)	2 (9.5%)	19 (7.5%)	*P* > 0.05
Flap (%)	0 (0%)	6 (6.1%)	0 (0%)	4 (12.9%)	6 (9.1%)	1 (4.8%)	17 (6.7%)	*P* > 0.05
Bucket-handle (%)	0 (0%)	2 (2.0%)	0 (0%)	6 (19.4%)^b^	5 (7.6%)	5 (15.6%)	18 (7.1%)	*P* < 0.05
Horizontal (%)	1 (14.3%)	3 (3.0%)	1 (5.9%)	0 (0%)	7 (10.6%)	2 (6.3%)	13 (5.2%)	*P* > 0.05
Oblique (%)	0 (0%)	3 (3.0%)	1 (5.9%)	1 (3.2%)	6 (9.1%)	1 (3.1%)	12 (4.8%)	*P* > 0.05
Complex (%)	0 (0%)	9 (9.1%)	0 (0%)	1 (3.2%)	13 (19.7%)	7 (21.9%)^c^	30 (11.9%)	*P* < 0.05

^a^*p* < 0.05, compared with Schatzker I, III, IV, V, VI

^b^*p* < 0.05, compared with Schatzker I, II, III, V

^c^*p* < 0.05, compared with Schatzker I, II, III, IV

^d^*p* < 0.05, compared with other patterns of meniscal tears

## Discussion

TPFs are increasingly common injuries involving intra-articular fractures concomitant with various patterns of meniscal tears, resulting in severe consequences and complications if not treated properly [19, 20]. Concomitant meniscal tears with tibial plateau fractures have been widely reported in several prior studies using arthroscopy to confirm the frequency of meniscal tears; nevertheless, most of these case series included only a small number of patients. The frequency of meniscal tears associated with TPFs to be within the range of approximately 23% to 57% [14, 21–23]. Fowble et al. [24] reported 11 meniscal tears (100% rate of lateral meniscal tears) in 23 TPFs diagnosed by arthroscopy and conventional open procedures intraoperatively. Simultaneously, Vangsness et al. [9] identified 17 meniscal tears in 36 TPFs diagnosed by arthroscopy, mainly in the lateral meniscal tears with frequency of 36%. An arthroscopic evaluation by Hung et al. [22] reported a 23% frequency of meniscal tears. A more recent large series in 98 cases using arthroscopy identified a 57% frequency of meniscal tears in patients with operative tibial plateau fracture [21].

This present study is the largest arthroscopy cohorts of operative TPFs to date, we found a higher overall frequency of meniscal tears which account for 66.7% (168 of 252) than has been previously reported. Furthermore, it is notable that the lateral meniscal tears were more susceptible to trauma as well with a frequency of 33.3% (84 of 252), which is comparable to previously reported series. As proposed by Schatzker et al. [25], meniscal tears are particularly common on the same side as the fracture, which may account for the higher frequency of lateral meniscal tears due to the highest proportion with lateral TPFs in this series. Furthermore, our results have reported the most frequent meniscal lesions with TPFs occurs to the lateral meniscus, followed by the bilateral meniscus and medial meniscus. It is known that Schatzker II is defined as a lateral tibial plateau split combined with articular depression. Actually, the injury force mechanism in Schatzker II has a potential diagnostic value in predicting meniscal tears. The present study identified that lateral meniscal tears were found to be strong associated with the Schatzker II. These findings could be explained by the fact that the injury mechanism of Schatzker II is characterized by axial combined with valgus loading forces applied to the knee flexion or extending position at the time of injury, which results in combined forces into the lateral meniscus within the knee joint. Consequently, it takes combined forces, including valgus and axial, and even shear and rotational forces, to bring about lateral meniscal tears in tibial plateau fractures.

In addition, it is noted that MRI examinations have also been used to assess meniscal tears in the setting of TPFs due to its excellent spatial resolution of soft-tissue injuries in the knee. Several studies have determined a high frequency of meniscal tears associated with TPFs diagnosed by MRI [7, 10]. Barrow et al. [26] found that a rate of 87% meniscal tears in 31 fractures. A previously study by Gardner et al. [7] in 103 patients with operative fractures demonstrated a 76% rate of LMT, as well as 44% MMT. Kode et al. [27] evaluated 22 TPFs through MRI and reported a 55% frequency of overall meniscal tears, with 41% in LMT and 14% in MMT. However, these reports using MRI served as a preoperative assessment of the meniscal tears in operative TPFs and may overstate the frequency of meniscal tears when compared to intraoperative arthroscopy findings [28]. Recently, Yan et al. [29] reported 27 patients with Schatzker IV TPFs using MRI, 81.5% patients had meniscal tears, 63.0% patients had LMT, and 44.4% patients had MMT. In our series of 243 TPFs, however, injury to lateral meniscus was 33.3% and to medial meniscus was 10.3%, which appeared less frequently than previously reported results, indicating that MRI examination potentially overestimating the true prevalence of meniscal tears associated with TPFs.

Complexity of injury mechanism and a combination of shearing and axial compression forces at the time of injury, meniscal lesions are often concurrent with TPFs. Although surgeons have traditionally relied on physical exam or MRI for detection of meniscal lesions, orthopedists have increasingly accepted arthroscopy as playing a critical role in preoperative assessment [30–32]. It is apparent to us that arthroscopic examination offers a reliable, rapid, accurate, and minimally invasive procedure in diagnosing meniscal tears especially concomitant with TPFs from Schatzker I to VI classification. With the use of our published literature and the assistance of thorough arthroscopy examination [33], we obtained an excellent view of intra-articular structures and encountered no complications, thus enabling accurate diagnosis and treatment of meniscal tears. All of the 243 patients in the current study were received by immediate arthroscopic examination after the internal fixation of tibial plateau fracture, and associated frequency and specific patterns of meniscal tears were accurately diagnosed during the surgery. However, previous reports in the literature have not analyzed the association between the associated frequency of meniscal tears and Schatzker classification in a large number of patients. Analysis of our data revealed that Schatzker II fracture was the most common type of tibial plateau fracture, and that it occurred at a significantly higher prevalence than Schatzker I, III, IV-VI. In our series, 168 of 243 patients with tibial plateau fracture had meniscal tears. Associated frequency of overall meniscal tears was noted to occur at a high rate in each Schatzker classification. We found no significant difference between the frequency of overall meniscal tears and Schatzker classification.

More importantly, previous reports in the literature did not investigate the association between patterns of meniscal tears and each Schatzker classification. Specific meniscal tears were associated with each type of tibial plateau fracture. Moreover, a good recognition and comprehensive understanding of the association between patterns of meniscal tears and Schatzker classification is critical to devise an appropriate surgical protocol, which aids to prepare surgeons to provide optimal treatment. Abdel-Hamid et al. [21] reported only 3 patterns of meniscal tears and peripheral tear of the meniscal tears in 38% of patients, which is the most common patterns of meniscal lesions according to the arthroscopy findings intra-operatively, but they did not analyze the relationship between meniscal tears morphology and Schatzker classification. Our series demonstrated statistically significant differences in the morphology of meniscal tears associated with different Schatzker classification. The most common patterns of meniscal tears in our study based on Schatzker classification were longitudinal tears. Furthermore, our results also identified that a significantly higher frequency of complex tears, which appeared most frequently in bilateral side lesions of meniscus and was more commonly observed in Schatzker VI and Schatzker V when compared to other Schatzker classification.

Some limitations must be acknowledged to this retrospective study. First of all, we did not report on clinical outcomes of meniscal tears treated with the assistance of an arthroscopy. The aim of our study, however, is not aimed at analyzing the clinical outcomes but only to describe and reveal the association between meniscal tears and TPFs. Second, the incidence rate of meniscal tears found at arthroscopy in this study may not have been exactly reflecting the true incidence in TPFs. Because patients may sustain meniscal tears at the ahead of trauma in the absence of knee pain. Furthermore, nondisplaced fractures and TPFs treated by ORIF without arthroscopy were not included in this literature. Despite these limitations, our study included a relatively large number of patients with TPFs following arthroscopy, which may result in increased power as compared to prior studies. Meanwhile, this evaluation allows the identification association between the prevalence of meniscal tears and TPFs as well as meniscal tear patterns and associated TPFs, ultimately aiding in treatment protocols.

## Conclusion

In conclusion, meniscal tears were commonly concomitant with tibial plateau fractures from Schatzker I to VI. Based on our series, we suggest that arthroscopic examination remains necessary for the intra-operative detection of meniscus integrity in tibial plateau fractures.

## Data Availability

All the data and material involving this article will be available upon request by sending an e-mail to the first author.

## Abbreviations

TPFs: Tibial plateau fractures
LMT: Lateral meniscal tears
MMT: Medial meniscal tears
BMT: Bilateral meniscal tears
ORIF: Open reduction and internal fixation
CRIF: Closed reduction and internal fixation
MRI: Magnetic resonance imaging
CT: Computer tomography
